# Evaluating service needs for veno-venous extracorporeal membrane oxygenation in patients with severe acute respiratory distress syndrome in Saskatchewan

**DOI:** 10.1038/s41598-023-45013-6

**Published:** 2023-10-17

**Authors:** Barsa Saha, Savannah Drapak, Jonathan F. Mailman, Sandy Kassir, Eric Sy

**Affiliations:** 1 School of Public Health, University of Saskatchewan, 107 Wiggins Rd, Saskatoon, SK S7N 5E5 Canada; 2 School of Health Sciences, Saskatchewan Polytech, 4635 Wascana Pkwy, Regina, SK S4P 3A3 Canada; 3 Pharmacy Department, Royal Jubilee Hospital, Vancouver Island Health Authority, Victoria, BC V8R 1J8 Canada; 4 College of Medicine, University of Saskatchewan, 1440-14th Avenue, Regina, SK S4P 0W5 Canada; 5 Faculty of Pharmaceutical Sciences, University of British Columbia, Vancouver, BC V6T 1Z3 Canada; 6 Research Department, Saskatchewan Health Authority, Wascana Rehabilitation Centre, 2180-23 Ave, Regina, SK S4S 0A5 Canada; 7 Department of Critical Care, Saskatchewan Health Authority, 1440-14th Avenue, Regina, SK S4P 0W5 Canada; 8 Surgical Intensive Care Unit, Regina General Hospital, 1440-14 Avenue, Regina, SK S4P 0W5 Canada

**Keywords:** Cardiology, Health care, Medical research

## Abstract

To determine the number of patients with acute respiratory distress syndrome (ARDS) who would be eligible to receive veno-venous extracorporeal membrane oxygenation (VV-ECMO). We conducted a retrospective observational study of ARDS patients admitted to Regina General Hospital Intensive Care Unit (ICU). VV-ECMO eligibility was assessed using selection criteria from the Extracorporeal Membrane Oxygenation for Severe Acute Respiratory Syndrome trial (EOLIA), the Extracorporeal Life Support Organization (ELSO), New South Wales (NSW), Critical Care Services Ontario (CCSO) and a Regina-restrictive criteria. Of 415 patients admitted between October 16, 2018, and January 21, 2021, 103 (25%) had mild, 175 (42%) had moderate, and 64 (15%) had severe ARDS. Of the cohort, 144 (35%) had bacterial pneumonia, 86 (21%) had viral pneumonia (including COVID-19), and 72 (17%) had aspiration pneumonia. Using the EOLIA, ELSO, NSW, CCSO and Regina-restrictive criteria, 7/415 (1.7%), 6/415 (1.5%), 19/415 (4.6%), 26/415 (6.3%) and 12/415 (2.9%) were eligible for VV-ECMO, respectively. Of all ECMO-eligible patients, only one (2.4%) actually received VV-ECMO, 20/42 (48%) received prone positioning and 21/42 (50%) received neuromuscular blockade. There is potential for service expansion of VV-ECMO in Regina; however, there is still a need to improve the delivery of evidence-based ARDS therapies.

## Introduction

Acute respiratory distress syndrome (ARDS) is an inflammatory syndrome^1^, which develops under several clinical conditions such as bacterial and viral pneumonia, including coronavirus-2019 disease (COVID-19)^2^. In an international prospective study, LUNG SAFE, 10% of all admissions to the intensive care unit (ICU) had ARDS, with a hospital mortality of 40%^3^. Common ventilation strategies and adjunctive therapies for ARDS include low tidal volume ventilation, optimizing positive end expiratory pressure (PEEP), prone positioning, neuromuscular blockade, and inhaled nitric oxide (iNO).

Recently, veno-venous extracorporeal membrane oxygenation (VV-ECMO) has been used as a rescue strategy for severe ARDS, based on a number of clinical trials and subsequent meta-analyses^4–7^. In an individual patient data meta-analysis of patients with severe ARDS, patients treated with VV-ECMO were found to have a relative risk of 0.75 (95% CI, 0.6–0.94) for 90-day mortality, compared to patients who did not receive VV-ECMO^5^. Consequently, the use of VV-ECMO for severe ARDS has been on the rise worldwide, and particularly with the recent COVID-19 pandemic^8^. In fact, in an international cohort study of the Extracorporeal Life Support Oxygenation (ELSO) registry, 1035 COVID-19 patients were supported with ECMO in 36 countries between January and May of 2020^9^.

Due to an increase in ECMO demand during the COVID-19 pandemic, an ECMO working group was formed at our hospital. Previously, we had assessed potential need for extracorporeal cardiopulmonary resuscitation need at our centre^10^. As such, the purpose of the study was to assess potential VV-ECMO volume in Regina, Saskatchewan to allow for program planning and improve patient outcomes.

## Methods

### Study design and setting

A retrospective observational study was conducted of consecutive ICU admissions with ARDS at Regina General Hospital (RGH) from October 16, 2018, to January 21, 2021. RGH is a tertiary care university-affiliated teaching hospital which can provide both VV- and veno-arterial (VA)-ECMO on an ad hoc basis. The hospital is a major referral centre in Southern Saskatchewan, serving about 500,000 residents in an area^11^ over 100,000 km^2^. RGH has a standard ventilation protocol with most patients receiving pressure-regulated volume control as the initial set mode, with a set tidal volume of 6–8 mL/kg predicted body weight, while maintaining the peak inspiratory pressure (PIP) ≤ 35 cm H_2_O and plateau pressure ≤ 30 cm H_2_O. PEEP may be set by static compliance measurements and/or esophageal pressure manometry.

### Participants

Patients were included if they met the following criteria: ≥ 18 years of age, received mechanical ventilation, and were diagnosed with ARDS based on the Berlin definition^1^. Patients who experienced respiratory failure primarily due to congestive heart failure were excluded.

### Covariates

Demographic and clinical information were collected, including age, biological sex, height, weight, comorbidities, oxygenation and ventilation parameters, use of adjunctive therapies, and information related to the potential contraindications such as severe bleeding, cardiac arrest prior to ECMO, severe immunosuppression, and poor neurological prognosis. Largest set and actual delivered tidal volumes (in mL) on the first day of mechanical ventilation were determined from tidal volume and spontaneous tidal volume measurements. The Charlson comorbidity index (CCI) and sequential organ failure assessment (SOFA) score were determined based on collected data^12,13^. We categorized patients into mild, moderate and severe ARDS based on the Berlin definition of ARDS^1^ (Table 1). Data was stored in a secure REDCap (Vanderbilt University, United States) database.

**Table 1:** Distribution of ECMO-eligible patients (under any criteria) across different severities of ARDS based only on day 1 PaO_2_/FiO_2_ ratio.

ARDS severity as of day 1 of mechanical ventilation	Eligible for VV-ECMO, N (%)	Non-eligible for VV-ECMO, N (%)	Total
No ARDS yet	2 (4.8%)	71 (19.0%)	73 (17.6%)
Mild, PaO_2_/FiO_2_: 200–300 mmHg	7 (16.7%)	96 (25.7%)	103 (24.8%)
Moderate, PaO_2_/FiO_2_: 100–199 mmHg	14 (33.3%)	161 (43.2%)	175 (42.2%)
Severe, PaO_2_/FiO_2_ < 100 mmHg	19 (45.2%)	45 (12.1%)	64 (15.4%)
Total	42	473	415

*ARDS* acute respiratory distress syndrome, *VV-ECMO* veno-venous extracorporeal membrane oxygenation.

### Veno-venous extracorporeal membrane oxygenation selection criteria

VV-ECMO eligibility was assessed using pre-defined selection criteria and contraindications that were derived from existing literature. The final criteria chosen included criteria from the Extracorporeal Membrane Oxygenation for Severe Acute Respiratory Syndrome (EOLIA) trial, ELSO, New South Wales (NSW), and CCSO (Table S2, Supplementary Appendix)^7,14–16^. The EOLIA trial is a well-known international randomized control trial of VV-ECMO in patients with severe ARDS^7^. The ELSO has previously published guidelines on ECMO management for COVID-19^14^. The more restrictive COVID-19 criteria were chosen over the more recent ELSO criteria as this study had taken place during the COVID-19 pandemic prior to the publication of the more recent criteria^17^. CCSO is a Canadian provincial body that has previously published referral criteria for ECMO provision^16^. The NSW criteria was chosen to represent criteria from another country from a well-established ECMO program^15^. The eligibility under each criterion was determined according to indications and only absolute contraindications (not relative contraindications). We assessed patients between days 1–14 of mechanical ventilation. A final ECMO criteria was created specific to RGH (Regina-restrictive) based on an internal modified Delphi study. Additional details pertaining to the Delphi study can be found in the Supplementary Appendix.

### Outcomes

The primary outcome was the number of patients that were ECMO-eligible and ECMO-ineligible based on the five different criteria during the study period. Secondary outcomes included in-hospital mortality, length of stay, and utilization of ventilation strategies and adjunctive therapies.

### Statistical analysis

All statistical analyses were conducted on Stata 17 (StataCorp, United States). Simple statistics were reported as frequency counts and percentages, means with standard deviations or medians with interquartile range (IQR) depending on the distribution of data. Normality testing was performed using the Shapiro–Wilk test. To compare proportions between eligible and non-eligible groups, χ^2^ squared or Fisher’s exact tests (for groups less than 10 counts) were used for categorical variables. *T* test or Wilcoxon rank sum tests were utilized for continuous variables depending on normality of data. To compare continuous variables between two or more groups, one-way ANOVA or Kruskal–Wallis H test were utilized also depending on normality.

### Ethics approval and consent to participate

The Research Ethics Board (REB) of the former Saskatchewan Health Authority had approved the study involving retrospective chart reviews (REB-21-17). A waiver of informed consent was obtained from the Saskatchewan Health Authority Research Ethics Board (REB-21-17). The University of Saskatchewan Behavioral Research Ethics Board approved the modified Delphi study and provided a certificate of approval (Beh-REB-2825). All research methods were carried out in accordance with the Health Information Protection Act (HIPA), the Tri-Council Policy Statement (TCPS 2) and the McMaster Chart Review Research Ethics.

## Results

### Full study cohort characteristics

**Figure 1:**
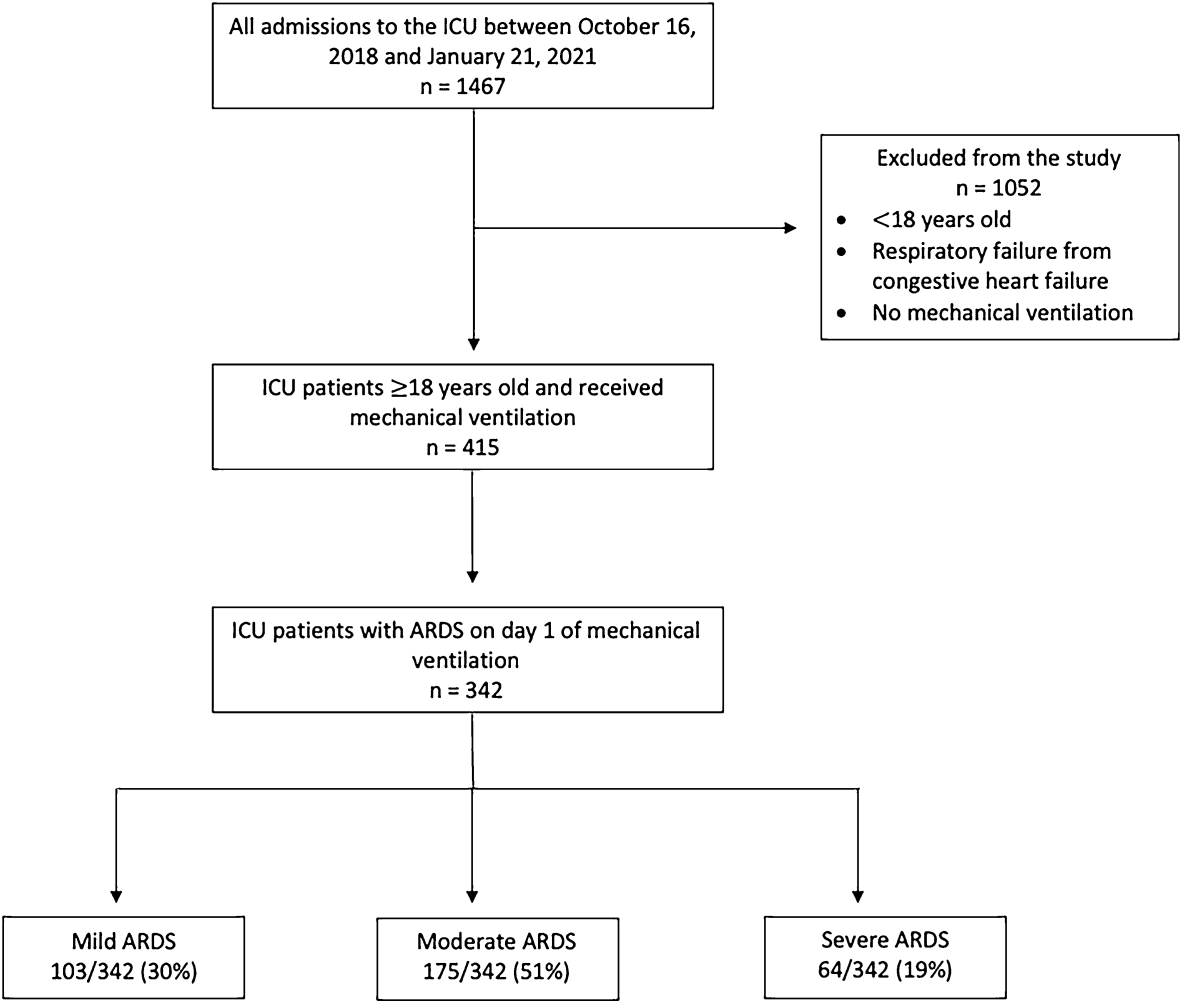
Flow diagram of the study.

Out of 1467 patients admitted to the RGH ICU, 415 patients were included in this study (Fig. 1). Of the 415 consecutive patients with ARDS, 165 (40%) patients were female, 144 (35%) had bacterial pneumonia, 86 (21%) had viral pneumonia (including COVID-19), 72 (17%) had aspiration pneumonia, 27 (6.5%) had trauma- or burn-related ARDS, 55 (13%) had other respiratory diagnoses and 30 (7.2%) had non-respiratory or chronic respiratory diagnoses. Forty-eight (12%) patients had COVID-19. According to the observed ventilator settings on day one of mechanical ventilation, patients received a median highest set tidal volume of 7.8 mL/kg (IQR 7.2–8.3), a median highest actual delivered tidal volume of 8.5 mL/kg (IQR 7.8–9.7), and a median PEEP of 10 cmH_2_O (IQR 8–14). In-hospital mortality occurred in 136/415 (33%) patients (Table 2). In the study, 342/415 (82%) patients met clinical parameters of ARDS by day one of mechanical ventilation.

**Table 2:** Demographics, clinical parameters and outcomes of ICU-admitted patients eligible for VV-ECMO under any criteria (EOLIA, ELSO, NSW, CCSO, Regina-restrictive) compared to patients who met neither criteria.

	Eligible for VV-ECMO (n = 42)	Non-eligible for VV-ECMO (n = 373)	p-value	All patients (n = 415)	Missing observations (N)
Age, median years (IQR)	65 (49–72)	60 (46–71)	0.25	61 (46–71)	0
Male gender, N (%)	33 (78.6%)	217 (58.2%)	0.01	250 (60.2%)	0
Charlson Comorbidity Index, median score (IQR)	1 (0–3)	2 (1–3)	0.33	1 (1–3)	0
Sequential Organ Failure Assessment, median score (IQR)	8 (6–10)	7 (5–9)	0.04	7 (5–9)	0
Clinical Frailty Score (IQR)	3 (2–4)	3 (3–4)	0.02	3 (3–4)	0
Body Mass Index, median kg/m^2^ (IQR)	29 (26–36)	28 (23–34)	0.09	28 (23–34)	0
Need for dialysis, N (%)	5 (11.9%)	17 (4.6%)	0.06	22 (5.4%)	5
COVID-19, N (%)	7 (16.7%)	41 (11.0%)	0.30	48 (11.6%)	0
Set VT, median mL/kg (IQR)	7.6 (7.1–8.1)	7.8 (7.2–8.3)	0.35	7.8 (7.2–8.3)	12
Actual delivered VT, median mL/kg (IQR)	8.4 (7.8–10.1)	8.5 (7.8–9.8)	0.91	8.5 (7.8–9.7)	4
Prone positioning, N (%)	20 (47.6%)	38 (10.2%)	< 0.001	58 (14.0%)	0
Neuromuscular blockade, N (%)	21 (50.0%)	51 (13.7%)	< 0.001	72 (17.3%)	0
Nitric oxide, N (%)	5 (11.9%)	6 (1.6%)	< 0.001	11 (2.7%)	0
Hospital length of stay, median days (IQR)	17 (8–25)	14 (8–32)	0.73	14 (8–31)	0
ICU length of stay, median days (IQR)	11 (3–19)	6 (4–12)	0.07	7 (4–12)	0
In-hospital mortality, N (%)	23 (54.8%)	113 (30.3%)	0.001	136 (32.8%)	0

*CCSO* Critical Care Services Ontario, *COVID-19* Coronavirus-19, *ELSO* Extracorporeal Life Support Organization, *EOLIA* Extracorporeal Membrane Oxygenation for Severe Acute Respiratory Syndrome, *ICU* intensive care unit, *IQR* interquartile range, *NSW* New South Wales, *VT* tidal volume, *VV-ECMO* veno-venous extracorporeal membrane oxygenation.

### Assessment of ECMO eligibility and associated characteristics

In this study, 42/415 (10%) patients were potentially eligible to receive VV-ECMO between all criteria. Between the five different criteria, 7/415 (1.7%), 6/415 (1.5%), 19/415 (4.6%), 26/415 (6.3%) and 12/415 (2.9%) patients were eligible for VV-ECMO, using the EOLIA, ELSO, NSW, CCSO and Regina-restrictive criteria, respectively (Table 3). Of all ECMO-eligible patients, only 1/42 (2.4%) patient received VV-ECMO and had met only the CCSO criteria for ECMO. Of all ECMO-eligible patients, 19/42 (45%) patients developed severe ARDS (Table 1). Further, a significantly large proportion of patients eligible for ECMO were males (79%) compared to non-eligible group (58%, p-value = 0.01). In terms of use of adjunctive therapies, 20/42 (48%) received prone positioning, 21/42 (50%) received neuromuscular blockade and 5/42 (12%) received iNO (Table S4, Supplementary Appendix).

**Table 3:** Demographics, clinical parameters and outcomes of ICU-admitted patients eligible for VV-ECMO by EOLIA, ELSO, NSW, CCSO and Regina-restrictive criteria.

	EOLIA criteria (n = 7)	ELSO criteria (n = 6)	NSW (n = 19)	CCSO criteria (n = 26)	Regina-restrictive criteria (n = 12)
Criteria met, N (%)	7 (1.7%)	6 (1.5%)	19 (4.6%)	26 (6.3%)	12 (2.9%)
Age, median years (IQR)	67 (54–70)	49 (31–69)	63 (44–73)	67 (54–73)	61 (52–70)
Male gender, N (%)	4 (57.1%)	6 (100%)	16 (84.2%)	19 (73.1%)	10 (83.3%)
Charlson Comorbidity Index, median score (IQR)	1 (0–3)	1.5 (1–3)	2 (0–5)	1 (0–2)	1 (0–2)
Sequential Organ Failure Assessment, median score (IQR)	10 (7–13)	11 (8–13)	8 (7–11)	8 (5–10)	8 (6–9)
Clinical Frailty Score (IQR)	3 (2–4)	2 (2–2)	3 (2–3)	3 (2–4)	3 (3–4)
Body Mass Index, median kg/m^2^ (IQR)	29 (26–31)	32 (30–46)	28 (24–32)	30 (28–41)	29 (27–35)
Need for dialysis, N (%)	2 (28.6%)	0 (0%)	3 (15.8%)	3 (11.5%)	0 (0%)
COVID-19, N (%)	1 (14.3%)	3 (50.0%)	2 (10.5%)	5 (19.2%)	4 (33.3%)
Set VT, median mL/kg (IQR)	7.7 (7.4–8.2)	7.4 (6.8–7.7)	7.3 (7.0–7.6)	7.8 (7.3–8.6)	7.0 (6.0–7.9)
Actual delivered VT, median mL/kg (IQR)	8.2 (8.0–8.9)	8.7 (7.0–10.4)	8.8 (7.2–10.2)	8.6 (8.0–10.4)	8.6 (6.4–10.4)
Prone positioning, N (%)	3 (42.9%)	5 (83.3%)	9 (47.4%)	12 (46.2%)	7 (58.3%)
Neuromuscular blockade, N (%)	4 (57.1%)	5 (83.3%)	10 (52.6%)	12 (46.2%)	8 (66.7%)
Nitric oxide, N (%)	2 (28.6%)	1 (16.7%)	2 (10.5%)	3 (11.5%)	2 (16.7%)
Hospital length of stay, median days (IQR)	8 (1–10)	1 (1–9)	12 (2–25)	19 (10–25)	15 (9–20)
ICU length of stay, median days (IQR)	3 (1–10)	1 (1–9)	8 (1–13)	13 (3–22)	13 (9–18)
In-hospital mortality, N (%)	6 (83.7%)	5 (83.3%)	12 (63.2%)	14 (53.9%)	9 (75.0%)

*CCSO* Critical Care Services Ontario, *COVID-19* Coronavirus-19, *ELSO* Extracorporeal Life Support Organization, *EOLIA* Extracorporeal Membrane Oxygenation for Severe Acute Respiratory Syndrome, *ICU* intensive care unit, *IQR* interquartile range, *NSW* New South Wales, *VT* tidal volume, *VV-ECMO* veno-venous extracorporeal membrane oxygenation.

Among all patients, there were 48 patients with COVID-19, of which seven (14.6%) were eligible for VV-ECMO, compared to 35 (9.5%) of patients who did not have COVID-19 (p = 0.30) (Table S5, Supplementary Appendix).

### Outcomes of ECMO-eligible patients

Although 279/415 (62%) of all patients survived to hospital discharge, only 1/7 (14%), 1/6 (17%), 7/19 (37%), 12/26 (46%), 4/12 (33%) patients, who met the EOLIA, ELSO, NSW, CCSO and Regina-restrictive criteria respectively, survived to hospital discharge. For all ECMO-eligible patients, in-hospital mortality was 54% (23/42) and the hospital length of stay was a median of 17 days (IQR 8–25). Additional results can be found in the Supplementary Appendix.

## Discussion

In this study, we used several different criteria varying in their level of stringency to evaluate potential ECMO need in our centre. Approximately six (1.5%) to 26 (6.3%) patients were eligible for VV-ECMO over a duration of 2.3 years at our centre, but only one patient received treatment with ECMO during that period. Thus, the estimated VV-ECMO volume at RGH could range from at least three to eleven cases of VV-ECMO per year. The estimated incidence of VV-ECMO use for ARDS would be 0.6 to 2.2 cases per 100,000 population per year (based on RGH’s catchment of approximately 500,000 residents)^11^. Statistics Canada forecasts a 14.3% to 43.8% growth in the population of Saskatchewan in the next 20 years^18^. With a potential rise in RGH’s catchment of 570,000 to 720,000 residents, an incidence of 0.7 to 3.2 cases per 100,000 population per year could be expected for VV-ECMO use for ARDS in 20 years.

We did attempt to distinguish between the need for ECMO among patients that had COVID-19 compared to patients that did not. Even if we were to exclude patients with COVID-19, we would expect around 0.3 to 1.8 cases of ECMO per 100,000 population per year. While it is difficult to predict when the next pandemic may occur, it is likely that a future pandemic may increase ECMO demand.

As such, an increase in local ECMO volume for patients with severe ARDS could be anticipated. For this to occur, human resources and resource allocation (i.e., trained perfusionists) would need to be assessed at RGH. Although ECMO services may be increased in Regina, Saskatchewan, current ECMO volume may be insufficient to maintain significant expertise (not withstanding additional patients who may be receiving VA-ECMO for cardiogenic shock and/or extracorporeal cardiopulmonary resuscitation).

In a prior study, higher annual adult ECMO centre volume was associated with lower mortality^19^. Previously, it has been suggested that centres should maintain an ECMO volume of at least 12 to 20 cases per year to optimize outcomes and maintain expertise^19^. However, recent evidence during the COVID-19 pandemic would suggest that newly formed ECMO centres may have acceptable outcomes when supervised in conjunction with an experienced centre^20^. In Japan, the Tokyo Medical and Dental University Hospital performs only 5 to 10 VV-ECMO cases per year, yet they were able to achieve a high survival rate with a multidisciplinary team approach^21^.

Therefore, there are several potential solutions including centralization of ECMO services in Saskatchewan to one tertiary care site and/or partnership with other provincial ECMO programs (i.e., Alberta Health Services and/or Manitoba Health). However, geographic, climate, and jurisdictional considerations in Saskatchewan limit the ability to transport unstable patients over large distances and likely justify the current situation of two ECMO referral centres in Saskatchewan.

### Comparison to other studies

Around the world, there are differences in the predicted incidence of VV-ECMO. The annual rates of VV-ECMO use for ARDS varied from 0.5 to 1 per 100,000 population in France^22^, 2.4 per 100,000 in Germany^23^, and 9.8 per 100,000 in South Korea^8^. Thus, at around three to eleven cases of VV-ECMO per year, our centre would have comparable incidence of VV-ECMO per population.

When comparing ARDS outcomes to other studies, the in-hospital mortality of VV-ECMO-eligible patients in our study (55%) was higher compared to ARDS patients in the VV-ECMO arm of the EOLIA trial (36%)^7^. For reference, mortality in the control group of the EOLIA trial was 57% despite a 28% crossover to ECMO^7^. This result is unsurprising given the subsequent Bayesian re-analysis and meta-analyses demonstrating benefit to VV-ECMO use. However, this emphasizes a potential need to improve VV-ECMO access at our institution.

On the other hand, there was low adherence to evidence-based ARDS therapies in our cohort, including low tidal volume ventilation, prone positioning, and neuromuscular blockade. Consequently, ECMO needs could be overestimated if these therapies were instituted routinely. For comparison to other ECMO-eligible patients in our study, ARDS patients participating in the EOLIA trial had lower tidal volume (mean 6.0 mL/kg in EOLIA trial versus median 8.4 mL/kg in our study), higher use of prone positioning (56% versus 48% in our cohort), higher use of iNO (51% versus 12%), and higher use of neuromuscular blockade (92% versus 50%)^7^.

### Future directions

Our study has important findings and implications for local quality improvement. This may include interventions to improve recognition of ARDS, improve adherence to evidence-based ARDS therapies, educate staff, develop local treatment guidelines, and streamline the referral process for VV-ECMO. In an observational before and after study, the implementation of an ARDS protocol reduced patient mortality by 12%, improved clinician recognition of ARDS, improved the detection of unsafe tidal volumes and airway pressures, and increased the use of ventilation strategies^24^.

## Limitations

Our study has limitations. First, we did not collect information on driving pressure, measures of respiratory effort such as P_0.1_ or airway occlusion pressure, or other measures of lung stress and strain, as these criterion had not previously been included in previously published ECMO inclusion or exclusion criterion^25^. Second, the retrospective nature of the study makes it difficult to accurately assess for contraindications, as they may not have been charted adequately and clinician judgement may also have played a role. Third, as there was only one patient who received ECMO in our cohort, patient outcomes of ECMO treatment could not be studied. Finally, as this is a single centre study, our findings may not necessarily apply to other centres.

## Conclusion

There may be potential need for expansion of VV-ECMO services in Saskatchewan. However, the use of low tidal volume ventilation, prone positioning, and neuromuscular blockade in patients with severe ARDS could be optimized through quality improvement, staff education, and protocolized care.

### Supplementary Information

Supplementary Information.

## Data Availability

The data that support the findings of this study may be available on request from the corresponding author and written permission from the Saskatchewan Health Authority. The data are not publicly available due to privacy and confidentiality restrictions from the Saskatchewan Health Authority.

## Abbreviations

ARDS: Acute respiratory distress syndrome
ICU: Intensive care unit
PEEP: Positive end expiratory pressure
iNO: Inhaled nitric oxide
FiO_2_: Fraction of inspired oxygen
PaO_2_: Partial pressure of oxygen
PIP: Peak inspiratory pressure
VV-ECMO: Veno-venous extracorporeal membrane oxygenation
VA-ECMO: Veno-arterial extracorporeal membrane oxygenation
RGH: Regina General Hospital
SOFA: Sequential organ failure assessment
CCI: Charlson Comorbidity Index
EOLIA: Extracorporeal Membrane Oxygenation for Severe Acute Respiratory Syndrome
ELSO: Extracorporeal Life Support Organization
CCSO: Critical Care Services Ontario
CESAR: Conventional Ventilatory Support VS Extracorporeal Membrane Oxygenation for Severe Adult Respiratory Failure
IQR: Interquartile range
