# Supplementary material for: Evaluating service needs for veno-venous extracorporeal membrane oxygenation in patients with severe acute respiratory distress syndrome in Saskatchewan
Source: Sci Rep. 2023 Oct 17;13:17627. doi: 10.1038/s41598-023-45013-6 (PMC10582080; doi:10.1038/s41598-023-45013-6)
Supplement: Supplementary file 1 — Supplementary Information. [file 41598_2023_45013_MOESM1_ESM.docx]

**Supplementary Appendix**

**Supplementary Methods**

*Modified-Delphi study and Regina-restrictive criteria*

A modified Delphi study to determine local criteria for veno-venous extracorporeal membrane oxygenation (VV-ECMO) was approved by the University of Saskatchewan Behavioral Research Ethics Board (Beh-REB-2825). Participants for this study included fifteen members of the Regina General Hospital (RGH) ECMO Working Group, including intensivists, ICU nurses, perfusionists, and cardiothoracic surgeons (**Table S1).** Through the use of surveys, we asked participants to state their relative agreement to various indications and contraindications for VV-ECMO, including oxygenation, carbon dioxide and mechanical ventilation criteria, as well as relative and absolute contraindications. Proposed criteria were determined a prior through a preliminary literature search. We searched PubMed, Cochrane Library, and Ovid MEDLINE databases, using the following MeSH terms: “Respiratory Distress Syndrome” and “Extracorporeal Membrane Oxygenation.” After reviewing relevant articles and guidelines, we used established criteria from the EOLIA trial (1), the ELSO guidelines (2), the CESAR trial (3), the Italian Ministry of Health (4), and others (see **Tables S2** and **S3**) (5–8). Agreement was assessed using a five-point Likert scale, and consensus of 75% was to be reached in every round for a criterion to be approved. Criteria that were not approved were modified and submitted for another round of voting, based on participant feedback. At the end of the third round, all statements having reached 75% consensus and associated rankings were used to determine the final eligibility criteria restrictive to Regina.

**Supplementary Results**

*Modified Delphi Study and Regina-restrictive criteria*

Of fifteen members of the ECMO Working Group, eight chose to participate. Response rates for rounds one, two, and three were 8/8 (100%), 6/8 (75%), and 8/8 (100%) respectively. The final agreed upon Regina-restrictive criteria are presented in **Table S2**.

*Full study cohort characteristics*

ICU and hospital length of stay were 7 (interquartile range [IQR] 4-12) and 14 (IQR 8-31) respectively (**Table 1**). In the cohort, 72/415 (17%) received neuromuscular blockade, 58/415 (14%) received prone positioning, 11/415 (2.7%) received inhaled nitric oxide (iNO), and 1/415 (0.2%) received ECMO. Of patients meeting the definition of acute respiratory distress syndrome (ARDS), 103/342 (30%) patients had mild, 175/342 (51%) had moderate, and 64/342 (19%) had severe ARDS.

*ARDS severity and associated use of optimal ventilation and adjunctive therapies*

Based on initial ventilator settings on day one, patients with mild, moderate, and severe ARDS had initial set tidal volumes of 7.8 (7.1-8.4), 7.9 (7.2-8.4) and 7.7 (7.4-8.1) ml/kg of predicted body weight respectively (**Table S4**). However, according to actual tidal volume delivered on day one of mechanical ventilation, patients with mild, moderate, and severe ARDS had received tidal volumes of 8.5 (7.7-9.5), 8.5 (7.9-9.9) and 8.7 (8.0-10.0) ml/kg of predicted body weight respectively. Furthermore, patients with mild, moderate, and severe ARDS received positive end-expiratory pressure (PEEP) of 8 (7-10), 10 (8-14) and 14 (12-18) cm H_2_O respectively. In terms of adjunctive therapies, only 23/64 (36%) of severe ARDS cases received prone positioning, 24/64 (38%) received neuromuscular blockade and 7/64 (11%) received iNO (**Table S4**).

*Associated characteristics of ECMO eligible patients*

The median age between all ECMO-eligible patients and non-eligible patients did not differ significantly (65 years [IQR 49-72] versus 60 years [IQR 46-71], p-value=0.25) (**Table 1**). The median Charlson comorbidity index score for ECMO-eligible patients was 1 (IQR 0-3) compared to 2 (IQR 1-3) for non-eligible patients (p-value=0.33). The median sequential organ failure assessment (SOFA) score was 8 (IQR 6-10) in ECMO-eligible patients compared to 7 (IQR 5-9) in non-eligible patients (p-value=0.04).

*Ventilation parameters in ECMO-eligible patients*

The median tidal volume delivered per kilogram of total body weight were similar between the ECMO-eligible and non-eligible groups; however, PEEP, PaO_2_/FiO_2_ ratio, peak inspiratory pressures (PIP) and arterial pH significantly differed. The median PEEP was higher in ECMO-eligible patients with a median of 14 cmH_2_O (p-value<0.001), the median PIP was higher in ECMO-eligible group with a median of 35 cmH_2_O (p-value<0.001), PaO_2_/FiO_2_ ratio was lower in ECMO-eligible group with median of 106 mmHg (p-value<0.001) and pH on first day of mechanical ventilation was also lower with a median of 7.25 (p-value=0.002) (**Table S5**). ECMO-eligible patients experienced respiratory failure mostly due to viral pneumonia (10/42, 24%), bacterial pneumonia (11/42, 26%) and other respiratory diagnosis (11/42, 26%).

**Supplementary Material Tables**

**Table S1.** Composition of the ECMO Working Group at Regina General Hospital

| **Role** | **Number of individuals** |
| --- | --- |
| Intensivist | 4 |
| Critical Care Associate Physician | 1 |
| Cardiac Surgeon | 3 |
| Intensive Care Unit Nurse | 2 |
| Respiratory Therapist | 1 |
| Perfusionist | 2 |
| Administrator | 1 |
| Pharmacist | 1 |

**Table S2**. Selection Criteria and Contraindications for VV-ECMO

|  | **Extracorporeal Membrane Oxygenation for Severe Acute Respiratory Syndrome (EOLIA) Criteria** (1) | **Extracorporeal Life Support Organization (ELSO) Criteria** (2) | **ECMO Referral Program in New South Wales (Australia) Criteria** (5) | **Critical Care Services Ontario Criteria (6)** | **Regina-restrictive Criteria** |
| --- | --- | --- | --- | --- | --- |
| **Selection Criteria** | 1. PaO_2_/FiO_2_ <50 mmHg for >3 hours, despite optimal ventilation (i.e., FiO_2_ $\geq$0.80, VT = 6 ml/kg, PEEP $\geq$10 cm H_2_O) 2. PaO_2_/FiO_2_ <80 mmHg for >6 hours, despite optimal ventilation 3. Arterial pH <7.25 with pCO_2_ $\geq$60 mmHg for >6 hours (respiratory rate of 35 breaths/min and Pplat $\leq$32 cm H_2_O) | 1. PaO_2_/FiO_2_ <50 mmHg for >3 hours 2. PaO_2_/FiO_2_ <60 mmHg for >6 hours 3. Arterial pH <7.20 with pCO_2_ $\geq$80 mmHg for >6 hours | 1. PaO_2_/FiO_2_ <100 mmHg AND pCO_2_ >100 mmHg for >1 hour, despite optimal ventilation (including PEEP $\geq$10 cm H_2_O, prone positioning, iNO) 2. PaO_2_/FiO_2_ <60 mmHg, despite optimal ventilation 3. No contraindications for VV-ECMO | 1. PaO_2_/FiO_2_ <50 mmHg for >3 hours 2. PaO_2_/FiO_2_ <80 mmHg for >6 hours 3. PaCO_2_ $\geq$60 mmHg for > 6 hours with respiratory rate >35 breaths/min and Pplat >30 cm H_2_O (i.e., PIP <30 cm H_2_O) | 1. PaO_2_/FiO_2_ <80 mmHg for >6 hours, despite optimal ventilation (i.e., FiO_2_ ≥0.80, PEEP ≥10 cm H_2_O, Pplat ≥30 cmH_2_O with VT ≤6 mL/kg, driving pressure >15 cmH2O) and trial of adjunctive therapies (i.e., prone positioning, iNO, neuromuscular blockade) 2. PaO_2_/FiO_2_ <50 mmHg for >3 hours, despite optimal ventilation and trial of adjunctive therapies 3. Arterial pH <7.25 with pCO_2_ ≥60 mmHg for >6 hours (respiratory rate of 35 breaths/min and Pplat ≤32 cm H_2_O) |
| **Contraindications** | 1. Time on MV > 7 days 2. Ages <18 3. Pregnancy 4. BMI >45 kg/m^2^ 5. History of heparin-induced thrombocytopenia 6. Moribund patients (or SAPS-II >90) 7. Need for VA-ECMO 8. Coma following cardiac arrest 9. Inability to cannulate 10. Non-reversible neurologic injury 11. Chronic respiratory failure 12. Malignant disease with 5-year fatal prognosis 13. Decision to withhold life-sustaining therapies | Absolute contraindications   1. Advanced age 2. Clinical Frailty Score $\geq$3 3. MV > 10 days 4. Significant comorbidities (e.g., cirrhosis, dementia, advanced lung disease, severe PVD, metastatic cancer, end-stage diabetes) 5. Severe multiple organ failure 6. Uncontrolled bleeding, contraindications to coagulation 7. Severe neurologic injury (e.g., anoxic brain injury, stroke etc.) 8. Ongoing CPR   Relative contraindications   1. Ages $\geq$65 2. BMI $\geq$40 kg/m^2^ 3. Immunocompromised status 4. Need for high dose vasopressor 5. Advanced chronic systolic heart failure | Absolute contraindications:   1. Significant comorbidity (irreversible neurological condition, cirrhosis with ascites, encephalopathy, history of variceal bleeding, AIDS, active malignancy) 2. Severe pulmonary hypertension (mPAP >50 mmHg) 3. Severe right or left heart failure (EF <25%) 4. Cardiac arrest   Relative contraindications:   1. Age >65 2. Multiple organ failure 3. Multiple trauma with uncontrolled hemorrhage 4. High pressure, high FiO_2_ through intermittent positive pressure ventilation for >1 week | Absolute contraindications:   1. Disseminated malignancy 2. Severe brain injury 3. Prolonged CPR without adequate tissue perfusion 4. Severe chronic organ dysfunction (e.g., emphysema, cirrhosis) 5. Severe chronic pulmonary hypertension 6. Non-reversible advanced comorbidity (e.g., CNS damage or terminal malignancy)   Relative contraindications:   1. End-stage renal disease 2. BMI $\geq$40 kg/m^2^ or weight $\geq$125 kg 3. Ages <18 and >65 4. Contraindication to anticoagulation 5. MV >7 days | Absolute contraindications:   1. Poor neurological prognosis (i.e., intracranial hemorrhage, brain death, large stroke) 2. Any cardiac arrest >10 minutes prior to ECMO cannulation with uncertain neurological prognosis 3. Patient wishes that express limitations to care 4. Patient’s veins are too small for vessel cannulation 5. Life expectancy <5 years due to comorbid disease (i.e., congestive heart failure, COPD, diabetes)   Relative contradictions:   1. BMI >45 kg/m^2^ 2. MV ≥15 days, including time receiving non-invasive ventilation 3. SOFA score ≥16 (SOFA score predicts ≥95.2% mortality) 4. CCI ≥5 (21% 10-year estimated survival) 5. Advanced immunocompromised state (i.e., poorly controlled HIV, hematopoietic stem cell transplant, active chemotherapy) 6. Reduced functional status (CFS ≥6, Moderate Frailty) 7. Irreversible lung disease (i.e., COPD on home O_2_, COPD with cor pulmonale, IPF on home O_2_, IPF with cor pulmonale) |

**Abbreviations:** BMI = body mass index; CCI = Charlson comorbidity index; CFS = clinical frailty score; CNS = central nervous system; CPR = cardiopulmonary resuscitation; COPD = chronic obstructive pulmonary disease; EF = ejection fraction; FiO_2_ = fraction of inspired oxygen; IPF = idiopathic pulmonary fibrosis; iNO = inhaled nitric oxide; IPPV = intermittent positive pressure ventilation; mPAP = mean pulmonary artery pressure; MV = mechanical ventilation; PaO_2_ = partial pressure of oxygen; pCO_2_ = partial pressure of carbon dioxide; PEEP = positive end expiratory pressure; PIP = peak inspiratory pressure; Pplat = plateau pressure;

SaO_2_ = peripheral oxygen saturation; SAPS = Simplified Acute Physiology Score; SOFA score = sequential organ failure assessment; SvO_2_ = central venous oxygen saturation; VT = Tidal volume

**Table S3**: Selection Criteria and Contraindications for VV-ECMO from different studies and guidelines

| Study/Guidelines | Selection Criteria | Contraindications |
| --- | --- | --- |
| Critical Care Services Ontario (6) | 1. PaO_2_/FiO_2_ <50 mmHg for >3 hours 2. PaO_2_/FiO_2_ <80 mmHg for >6 hours 3. PaCO2 $\geq$60 mmHg for > 6 hours with respiratory rate >35 breaths/min and Pplat > 30 cm H_2_O (i.e., PIP < 30 cm H_2_O) | Absolute contraindications:   1. Disseminated malignancy 2. Severe brain injury 3. Prolonged CPR without adequate tissue perfusion 4. Severe chronic organ dysfunction (e.g., emphysema, cirrhosis) 5. Severe chronic pulmonary hypertension 6. Non-reversible advanced comorbidity (e.g., CNS damage or terminal malignancy)   Relative contraindications:   1. End-stage renal disease 2. BMI $\geq$ 40 kg/m^2^ or weight $\geq$125 kg 3. Ages <18 and >65 4. Contraindication to anticoagulation 5. MV > 7 days |
| CESAR  (3) | 1. Severe but potentially reversible ARDS 2. Murray score >3 3. Uncompensated hypercapnia with pH >7.2, despite optimal conventional therapy | 1. Ages <18 and >65 2. PIP >30 cm H2O or FiO2 >0.8 for >7 days 3. Intracranial bleeding 4. Contraindication to anticoagulation 5. Limitation of support |
| Prior nationwide trends for ECMO referral in the United States  (7) | 1. Severe oxygen impairment (PaO_2_/FiO_2_ <120-150 mmHg) 2. Periods of prolonged desaturations or elevated airway pressures, despite ventilation optimization 3. Considered on case-by-case basis by ECMO team | Absolute contraindications:   1. Ages >70 2. Multiorgan failure 3. Active malignancy 4. Hepatic disease 5. Severe neutropenia (neutrophil count <1000/mm^3^   Relative contraindications:   1. BMI > 40 kg/m^2^ 2. Active bleeding 3. Chronic renal dysfunction 4. Immune suppression 5. Concurrent infection with multi-drug resistant organisms |
| Chinese Society of Extracorporeal Life Support  (8) | 1. PaO_2_/FiO_2_ <100 mmHg when FiO_2_ =1.0 2. PaO_2_/FiO_2_ <50 mmHg for >3 hours 3. PaO_2_/FiO_2_ <80 mmHg for >6 hours 4. Arterial pH <7.2 with respiratory rate >35 breaths/min and Pplat > 30 cm H_2_O 5. Severe thoracic air leak syndrome 6. Cardiogenic shock or cardiac arrest | No absolute contraindications  Relative contraindications:   1. Drug immunosuppression (neutrophil count <400/mm^3^) 2. Inability to cannulate (due to reasons including malformations or lesions of blood vessels) 3. Need for VA-ECMO 4. Moderate to severe aortic valve insufficiency; acute aortic dissection 5. Multiple organ failure 6. Contraindications to anticoagulation 7. Comorbidity with unrecoverable diseases (e.g., advanced malignant tumors, severe CNS damage) 8. Need of MV for >7 days |
| ECMO referral program in New South Wales, Australia  (5) | Acute respiratory failure indications:   1. PaO_2_/FiO_2_ <100 mmHg AND pCO_2_ >100 mmHg for >1 hour, despite optimal ventilation (including PEEP $\geq$ 10 cm H_2_O, prone positioning, inhaled NO) 2. PaO_2_/FiO_2_ <60 mmHg, despite optimal ventilation 3. No contraindications for VV-ECMO | Absolute contraindications:   1. Significant comorbidity (irreversible neurological condition, cirrhosis with ascites, encephalopathy, history of variceal bleeding, AIDS, active malignancy) 2. Severe pulmonary hypertension (mPAP >50 mmHg) 3. Severe right or left heart failure (EF <25%) 4. Cardiac arrest   Relative contraindications:   1. Age >65 2. Multiple organ failure 3. Multiple trauma with uncontrolled hemorrhage   High pressure, high FiO_2_ IPPV for >1 week |
| ELSO  (2) | 1. PaO_2_/FiO_2_ <50 mmHg for >3 hours 2. PaO_2_/FiO_2_ <60 mmHg for >6 hours 3. Arterial pH <7.20 with pCO_2_ $\geq$ 80 mmHg for >6 hours | Absolute contraindications   1. Advanced age 2. Clinical Frailty Score $\geq$3 3. MV > 10 days 4. Significant comorbidities (e.g., cirrhosis, dementia, advanced lung disease, severe PVD, metastatic cancer, end-stage diabetes) 5. Severe multiple organ failure 6. Uncontrolled bleeding, contraindications to coagulation 7. Severe neurologic injury (e.g., anoxic brain injury, stroke etc.) 8. Ongoing CPR   Relative contraindications   1. Ages $\geq$ 65 2. BMI $\geq$ 40 kg/m^2^ 3. Immunocompromised status 4. Need for high dose vasopressor 5. Advanced chronic systolic heart failure |
| EOLIA  (1) | 1. PaO_2_/FiO_2_ <50 mmHg for >3 hours, despite optimal ventilation (i.e., FiO_2_ $\geq$0.80, VT = 6 ml/kg, PEEP $\geq$10 cm H_2_O) 2. PaO_2_/FiO_2_ <80 mmHg for >6 hours, despite optimal ventilation 3. Arterial pH <7.25 with pCO_2_ $\geq$ 60 mmHg for >6 hours (respiratory rate of 35 breaths/min and Pplat $\leq$32 cm H_2_O) | 1. Time on MV > 7 days 2. Ages <18 3. Pregnancy 4. BMI > 45 kg/m^2^ 5. History of heparin-induced thrombocytopenia 6. Moribund patients (or SAPS-II >90) 7. Need for VA-ECMO 8. Coma following cardiac arrest 9. Inability to cannulate 10. Non-reversible neurologic injury 11. Chronic respiratory failure 12. Malignant disease with 5-year fatal prognosis 13. Decision to withhold life-sustaining therapies |
| Italian Ministry of Health  (4) | 1. SaO_2_ <85% for >1 hour 2. Oxygenation Index >25 for >6 hours despite ventilation optimization (VT = 4-6 ml/kg, Pplat $\leq$30 cm H_2_O) 3. PaO_2_/FiO_2_ <100 mmHg for >6 hours with PEEP $\geq$ 10 cm H_2_O despite ventilation optimization 4. Hypercapnia with pH >7.25 5. SvO_2_ <65% with hematocrit >30 on vasoactive drug infusion | 1. If terminally ill with short life expectancy 2. Advanced and prolonged multiple organ dysfunction 3. Severe chronic lung disease |

**Abbreviations:** CESAR = Conventional Ventilatory Support VS Extracorporeal Membrane Oxygenation for Severe Adult Respiratory Failure Trial; COVID-19 = coronavirus-19 disease ; CNS = central nervous system; EF = ejection fraction; ELSO = Extracorporeal Life Support Organization; EOLIA = Extracorporeal Membrane Oxygenation for Severe Acute Respiratory Syndrome; FiO_2_ = fraction of inspired oxygen; IPPV = intermittent positive pressure ventilation; mPAP = mean pulmonary artery pressure; MV = mechanical ventilation; PaO_2_ = partial pressure of oxygen; pCO_2_ = partial pressure of carbon dioxide; PEEP = positive end expiratory pressure; PIP = peak inspiratory pressure; Pplat = plateau pressure; SAPS = Simplified Acute Physiology Score; BMI = body mass index; VA-EMCO = veno-arterial extracorporeal membrane oxygenation; SaO_2_ = peripheral oxygen saturation; SvO_2_ = central venous oxygen saturation;

**Table S4:** Comparison of ventilation parameters and adjunctive therapy use across different severity levels of ARDS, based on day one of mechanical ventilation

|  | No ARDS yet as of day one of ventilation (n=73) | Mild ARDS (n=103) | Moderate ARDS (n=175) | Severe ARDS (n=64) | p-value | Missing observations  (N) |
| --- | --- | --- | --- | --- | --- | --- |
| **Ventilation Parameters** | | | | | |  |
| Patients on MV, N (%) | 61 (83.6%) | 90 (87.4%) | 148 (85.6%) | 54 (84.5%) | 0.89 | 0 |
| PIP, median cm H_2_O  (IQR) | 24 (20-30) | 27 (22-32) | 33 (28-38) | 35 (30-41) | <0.001 | 0 |
| PEEP, median cm H_2_O  (IQR) | 8 (5-8) | 8 (7-10) | 10 (8-14) | 14 (12-18) | <0.001 | 2 |
| pH, median (IQR) | 7.35  (7.26-7.40) | 7.30  (7.22-7.38) | 7.29  (7.20-7.37) | 7.23  (7.16-7.30) | <0.001 | 3 |
| PaO_2_/FiO_2_, median (IQR) | 354  (325-400) | 240  (218-272) | 147  (120-174) | 75  (59-89) | <0.001 | 5 |
| Set VT, median ml/kg (IQR) | 7.8  (7.2-8.3) | 7.8  (7.1-8.4) | 7.9  (7.2-8.4) | 7.7  (7.4-8.1) | 0.99 | 12 |
| Actual delivered VT, median ml/kg (IQR) | 8.4  (7.7-9.3) | 8.5  (7.7-9.5) | 8.5  (7.9-9.9) | 8.7  (8.0-10.0) | 0.57 | 4 |
| **Adjunctive Therapies** | | | | | |  |
| Prone positioning,  N (%) | 2 (2.7%) | 6 (5.8%) | 27 (15.4%) | 23 (35.9%) | 0.001 | 0 |
| Neuromuscular blockade,  N (%) | 9 (12.3%) | 12 (11.7%) | 27 (15.4%) | 24 (37.5%) | 0.001 | 0 |
| Nitric oxide,  N (%) | 1 (1.4%) | 1 (1.0%) | 2 (1.1%) | 7 (10.9%) | 0.001 | 0 |
| Receipt of ECMO, N (%) | 0 (0%) | 1 (1.0%) | 0 (0%) | 0 (0%) | 0.58 | 0 |

**Abbreviations: ARDS =** Acute respiratory distress syndrome; FiO_2_  = Fraction of inspired oxygen; IQR = Interquartile range; MV = Mechanical ventilation; PaO_2_  = Partial pressure of oxygen; PEEP = Positive end expiratory pressure; PIP = Peak inspiratory pressure; VT = Tidal volume; VV-ECMO = Veno-venous extracorporeal membrane oxygenation

**Table S5:** Eligibility for extracorporeal membrane oxygenation based on COVID-19 status

| Criteria | COVID-19 positive  (n=48) | COVID-19 negative  (n=367) | p-value |
| --- | --- | --- | --- |
| EOLIA criteria | 1 (2.1%) | 6 (1.6%) | 0.58 |
| ELSO criteria | 3 (6.3%) | 3 (1%) | 0.02 |
| NSW criteria | 2 (4.2%) | 17 (4.6%) | 0.62 |
| CCSO criteria | 5 (10.4%) | 21 (5.7%) | 0.17 |
| Regina-restrictive criteria | 4 (8.3%) | 8 (2.2%) | 0.04 |
| Any criteria | 7 (14.6%) | 35 (9.5%) | 0.30 |

**Table S6:** Comparison of ventilation parameters and adjunctive therapy use between patients eligible for VV-ECMO under any criteria (EOLIA, ELSO, NSW, CCSO, Regina-restrictive) and patients who met neither criteria

|  | Eligible for  VV-ECMO  (n=42) | Non-eligible for  VV-ECMO  (n=373) | p-value | All patients  (n=415) | Missing observations (N) |
| --- | --- | --- | --- | --- | --- |
| **Ventilation Parameters** | | | | | |
| Patients on MV, N (%) | 35 (83.3%) | 318 (85.3%) | 0.74 | 353 (85.1%) | 0 |
| PEEP, median cmH_2_O (IQR) | 14 (10-16) | 10 (8-12) | <0.001 | 10 (8-14) | 0 |
| PIP, median cmH_2_O (IQR) | 35 (28-41) | 30 (25-36) | <0.001 | 30 (25-36) | 2 |
| pH, median (IQR) | 7.25 (7.17-7.30) | 7.30 (7.21-7.38) | 0.002 | 7.30 (7.20-7.38) | 3 |
| PaO_2_/FiO_2_, median (IQR) | 106 (59-195) | 184 (124-269) | <0.001 | 178 (116-260) | 5 |
| Set VT, median ml/kg (IQR) | 7.6 (7.1 - 8.1) | 7.8 (7.2 - 8.3) | 0.35 | 7.8 (7.2-8.3) | 12 |
| Actual delivered VT, median ml/kg (IQR) | 8.4 (7.8 – 10.1) | 8.5 (7.8 - 9.8) | 0.91 | 8.5 (7.8-9.7) | 4 |
| **Adjunctive Therapies** | | | | | |
| Prone positioning,  N (%) | 20 (47.6%) | 38 (10.2%) | <0.001 | 58 (14.0%) | 0 |
| Neuromuscular blockade,  N (%) | 21 (50.0%) | 51 (13.7%) | <0.001 | 72 (17.3%) | 0 |
| Nitric oxide,  N (%) | 5 (11.9%) | 6 (1.6%) | <0.001 | 11 (2.7%) | 0 |
| Receipt of ECMO, N (%) | 1 (2.4%) | 0 (0%) | 0.10 | 1 (0.2%) | 0 |

**Abbreviations:** CCSO = Critical Care Services Ontario; ELSO = Extracorporeal Life Support Organization; EOLIA = Extracorporeal Membrane Oxygenation for Severe Acute Respiratory Syndrome; FiO_2_  = Fraction of inspired oxygen; IQR = Interquartile range; MV = Mechanical ventilation; NSW = New South Wales; PaO_2_  = Partial pressure of oxygen; PEEP = Positive end expiratory pressure; PIP = Peak inspiratory pressure; VT = Tidal volume; VV-ECMO = Veno-venous extracorporeal membrane oxygenation

**Additional Appendix References**

1. Combes A, Hajage D, Capellier G, Demoule A, Lavoué S, Guervilly C, et al. Extracorporeal Membrane Oxygenation for Severe Acute Respiratory Distress Syndrome. N Engl J Med. 2018;378(21):1965–75.

2. Shekar K, Badulak J, Peek G, Boeken U, Dalton HJ, Arora L, et al. Extracorporeal Life Support Organization Coronavirus Disease 2019 Interim Guidelines: A Consensus Document from an International Group of Interdisciplinary Extracorporeal Membrane Oxygenation Providers. ASAIO J Am Soc Artif Intern Organs 1992. 2020 Jul;66(7):707–21.

3. Peek GJ, Mugford M, Tiruvoipati R, Wilson A, Allen E, Thalanany MM, et al. Efficacy and economic assessment of conventional ventilatory support versus extracorporeal membrane oxygenation for severe adult respiratory failure (CESAR): a multicentre randomised controlled trial. Lancet. 2009 Oct 17;374(9698):1351–63.

4. Ciapetti M, Cianchi G, Zagli G, Greco C, Pasquini A, Spina R, et al. Feasibility of inter-hospital transportation using extra-corporeal membrane oxygenation (ECMO) support of patients affected by severe swine-flu(H1N1)-related ARDS. Scand J Trauma Resusc Emerg Med. 2011 May 27;19:32.

5. Forrest P, Ratchford J, Burns B, Herkes R, Jackson A, Plunkett B, et al. Retrieval of critically ill adults using extracorporeal membrane oxygenation: an Australian experience. Intensive Care Med. 2011 May;37(5):824–30.

6. Critical Care Services Ontario. ECMO Consultation Guidelines: Guidance Document for Ontario Hospitals [Internet]. 2020 Jan [cited 2021 Aug 13]. Available from: https://criticalcareontario.ca/wp-content/uploads/2020/10/ECMO-Consultation-Guidelines_Guidance-Document-for-Ontario-Hospitals-FINAL-2020.pdf

7. Rush B, Wiskar K, Berger L, Griesdale D. Trends in Extracorporeal Membrane Oxygenation for the Treatment of Acute Respiratory Distress Syndrome in the United States. J Intensive Care Med. 2017;32(9):535–9.

8. Chinese Society of Extracorporeal Life Support. [Recommendations on extracorporeal life support for critically ill patients with novel coronavirus pneumonia]. Zhonghua Jie He He Hu Xi Za Zhi Zhonghua Jiehe He Huxi Zazhi Chin J Tuberc Respir Dis. 2020 Mar 12;43(3):195–8.
